# MUC (Memory, Unification, Control) and beyond

**DOI:** 10.3389/fpsyg.2013.00416

**Published:** 2013-07-12

**Authors:** Peter Hagoort

**Affiliations:** Donders Institute for Brain, Cognition and Behaviour, Max Planck Institute for Psycholinguistics, Radboud University NijmegenNijmegen, Netherlands

**Keywords:** neurobiology of language, Memory, Unification, Control, speaker meaning, language connectivity

## Abstract

A neurobiological model of language is discussed that overcomes the shortcomings of the classical Wernicke-Lichtheim-Geschwind model. It is based on a subdivision of language processing into three components: Memory, Unification, and Control. The functional components as well as the neurobiological underpinnings of the model are discussed. In addition, the need for extension of the model beyond the classical core regions for language is shown. The attention network and the network for inferential processing are crucial to realize language comprehension beyond single word processing and beyond decoding propositional content. It is shown that this requires the dynamic interaction between multiple brain regions.

## Introduction

**Figure 1:**
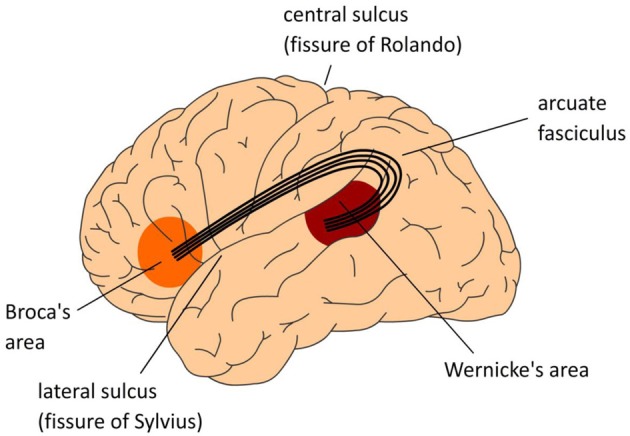
**The classical Wernicke-Lichtheim-Geschwind model of the neurobiology of language**. In this model Broca's area is crucial for language production, Wernicke's area subserves language comprehension, and the necessary information exchange between these areas (such as in reading aloud) is done via the arcuate fasciculus, a major fiber bundle connecting the language areas in temporal cortex (Wernicke's area) and frontal cortex (Broca's area). The language areas are bordering one of the major fissures in the brain, the so-called Sylvian fissure. Collectively, this part of the brain is often referred to as perisylvian cortex.

An adequate neurobiological model of our uniquely human language faculty has to meet the following two requirements: (1) it should decompose language skills such as speaking and listening into the contributing types of knowledge and processing steps (the cognitive architecture); (2) it should specify how these are instantiated in, and supported by the organization of the human brain (the neural architecture). Until not too long ago, the neurobiological model that has dominated the field was the Wernicke-Lichtheim-Geschwind (WLG) model (see Figure 1). In this model, the human language faculty was situated in the left perisylvian cortex, with a strict division of labor between the frontal and temporal regions. Wernicke's area in left temporal cortex was assumed to subserve the comprehension of speech, whereas Broca's area in left inferior frontal cortex was claimed to subserve language production. The arcuate fasciculus connected these two areas.

Even today, this model is still influential. For instance, in a recently published study (Moorman et al., 2012) one reads “Broca's area in the frontal lobe and Wernicke's area in the temporal lobe are crucially involved in speech production and perception, respectively.” (p. 12782). Many similar quotations can be found. Despite its impact until this very day, the classical model is wrong (cf. Poeppel et al., 2012). Although Broca's area, Wenicke's area and adjacent cortex are still considered to be key nodes in the language network, the distribution of labor between these regions is different than was claimed in the WLG model. Lesions in Broca's region are since long known to impair not only language production but also language comprehension (Caramazza and Zurif, 1976), whereas lesions in Wernicke's region also affect language production. More recently, neuroimaging studies provided further evidence that the classical view on the role of these regions is no longer tenable. For example, central aspects of language production and comprehension are subserved by shared neural circuitry (Menenti et al., 2011; Segaert et al., 2012). Moreover, the classical model focused on single word processing, whereas a neurobiological account of language processing in its full glory should also take into account what goes on beyond production and comprehension of single words. As a consequence of the mounting evidence against the classical WLG model, in recent years alternative neurobiological models for language have been proposed (e.g., Friederici, 2002; Hagoort, 2005; Hickok and Poeppel, 2007). Here I will focus mainly on the Memory-Unification-Control (MUC) model that I proposed in 2005 (Hagoort, 2005). After describing its three components, I will discuss the evidence that has accumulated in support of the model, and I will suggest extensions of the model on the basis of recent empirical evidence.

## Memory, unification, and control

The MUC model distinguishes three functional components of language processing: Memory, Unification and Control. The Memory component refers to the linguistic knowledge that in the course of language acquisition gets encoded and consolidated in neocortical memory structures. It is the only language-specific component of the model. The knowledge about the building blocks of language (e.g., phonological, morphological, syntactic building blocks) is domain specific and hence coded in a format that is different from, say, color and visual object information.

However, language processing is more than memory retrieval and more than the simple concatenation of retrieved lexical items. The expressive power of human language derives from the possibility to combine elements from memory in novel ways. In the model this process of deriving new and complex meaning from the lexical building blocks is referred to as Unification. Unification thus refers to the assembly of pieces stored in memory into larger structures, with contributions from context. Classically, psycholinguistic studies of unification have focused on syntactic analysis. But, crucially, unification operations take place not only at the syntactic processing level, but are a hallmark of language across representational domains (cf. Jackendoff, 2002, 2007). Thus, at the semantic and phonological levels, too, lexical elements are combined and integrated into larger structures. Hence I distinguish between syntactic, semantic and phonological unification (cf. Hagoort, 2005).

Finally, the Control component relates language to joint action and social interaction. Executive control is invoked, for instance, when the contextually appropriate target language has to be selected, for handling the joint action aspects of using language in conversational settings, for selecting the appropriate register in different social situations, etcetera. We will later see that languages also have built-in linguistic devices that trigger the attentional control system into operation.

**Figure 2:**
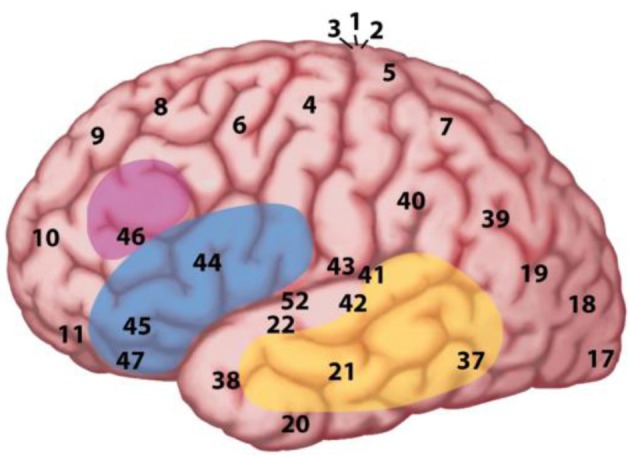
**The MUC model of language**. The figure displays a lateral view of the left hemisphere. The numbers indicate Brodmann areas. These are areas with differences in the cytoarchitectonics (i.e., composition of cell types). The memory areas are in the temporal cortex (in yellow) including the angular gyrus in parietal cortex. Unification requires the contribution of Broca's area (Brodmann areas 44 and 45) and adjacent cortex (Brodmann areas 47 and 6) in the frontal lobe. Control operations recruit another part of the frontal lobe (in pink), and the Anterior Cingulate Cortex (ACC; not shown in the figure), as well as areas involved in attention.

**Figure 3:**
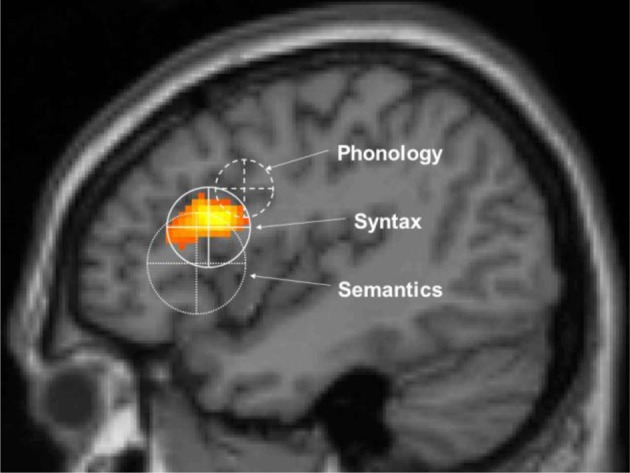
**The unification gradient in left inferior frontal cortex**. Activations and their distribution are shown, related to semantic, syntactic and phonological processing. Regions are based on the meta-analysis in Bookheimer. The centers represent the mean coordinates of the local maxima, the radii represent the standard deviations of the distance between the local maxima and their means. The activation shown is from artificial grammar violations in Petersson et al. (2004) (courtesy of Karl Magnus Petersson).

In the MUC model, the distribution of labor is as follows (see Figure 2): regions in the temporal cortex (in yellow) and the angular gyrus in parietal cortex subserve the knowledge representations that have been laid down in memory during acquisition. These regions store information including phonological word forms, morphological information, and the syntactic templates associated with noun, verbs, adjectives (for details, see Hagoort, 2003, 2005, 2009a,b). They also include semantic convergence zones, but on the whole conceptual knowledge is quite widely distributed (Binder and Desai, 2011). Dependent on knowledge type, different parts of temporal and parietal cortex are involved. Frontal regions (Broca's area and adjacent cortex; in blue) are crucial for unification operations. These operations generate larger structures from the building blocks that are retrieved from memory. Within left inferior frontal cortex (Unification Space), a spatial activation gradient is observed. The distribution of the activations seems to depend on the type of information that gets unified. Semantic unification recruits BA 47 and BA 45; syntactic unification has its focus in BA 45 and BA 44; phonological processes recruit BA 44 and ventral parts of BA 6 (see Figure 3). In addition, executive control needs to be exerted, such that the correct target language is selected, turn taking in conversation is orchestrated, the correct register is selected, attention is paid to the most relevant information in the input, and so forth. Control regions involve dorsolateral prefrontal cortex (in pink), and midline structure including the anterior cingulate cortex and the parts of parietal cortex that are involved in attention (not shown in Figure 2).

The distribution of labor in the MUC model is not absolute. Sometimes I have been misinterpreted as proposing a hypothesis that treats “natural language composition as monolithic and localized to a single region.” (Poeppel et al., 2012, p. 14310). This is incorrect. I hold the view that language functions do not reside in single brain regions. Instead, language is subserved by dynamic networks of brain regions, including the ones just outlined. Ultimately the mapping of a given language function onto the neural architecture of the brain is in terms of a network of brain areas instantiating that particular language function (Mesulam, 1998; McIntosh, 2008; Sporns, 2011; Turken and Dronkers, 2011). Typically, each node in such a network will participate dynamically in other functional networks as well. Although one can claim a certain contribution of a specific region (e.g., part of Broca's area), it is crucial to realize that such a contribution depends on the interaction with other regions that are part of the network. In short, “the mapping between neurons and cognition relies less on what individual nodes can do and more on the topology of their connectivity.” (Sporns, 2011, p. 184). Therefore, before discussing the empirical evidence for the distribution of labor within the MUC framework, I will discuss the connectivity profile of the language networks in the brain.

## The network topology of the language cortex

In the classical WLG model the arcuate fasciculus plays a central role in connecting the language-relevant parts of the brain. This fasciculus connects Broca's area and Wernicke's area, the two central nodes in the language network. It has become clear, however, that the language network in the left hemisphere is much more extended than was assumed in the classical model, and not only includes regions in the left hemisphere but also in the right hemisphere. However, the evidence of additional activations in the right hemisphere and areas other than Broca's and Wernicke's, does not take away the crucial role of left perisylvian cortex. In a recent meta-analysis based on 128 neuroimaging studies, Vigneau et al. (2010) compared left and right hemisphere activations observed in relation to language processing. On the whole, for phonological, lexico-semantic, and sentence or text processing, the number of activation peaks in the right hemisphere comprised less than one third of the activation peaks in the left hemisphere. Moreover in the large majority of cases the right hemisphere activations were found in homotopic regions, suggesting a strong inter-hemispheric dependency. It is therefore justified to think that for the large majority of the human population (e.g., with the exception of some portion of left-handers, cases of left hemispherectomy, etc.), the language-readiness of the human brain resides to a large extent in the organization of the left perisylvian cortex. One emerging generalization is that the network of cortical regions subserving output processing (production) is very strongly (left) lateralized; in contrast, the computational subroutines underlying comprehension appear to recruit both hemispheres rather more extensively, even though here too there exists compelling lateralization, especially for syntax (Menenti et al., 2011).

Moreover, the network organization of the left perisylvian cortex shows characteristics that distinguishes it from the right perisylvian cortex—and from homologue regions in other primates.

**Figure 4:**
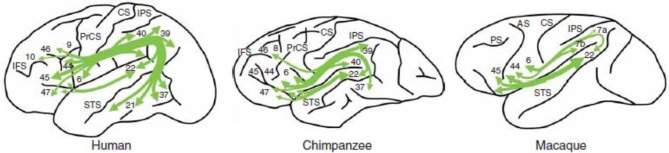
**The arcuate fasciculus in human, chimpanzee and macaque in a schematic lateral view of the left hemisphere**. From Rilling et al. (2008); courtesy of Nature Publishing Group.

A recent technique for tracing fiber bundles in the living brain is Diffusion Tensor Imaging (DTI). Using DTI, Rilling et al. (2008) tracked the arcuate fasciculus in humans, chimpanzees and macaques. These authors found in humans a prominent temporal lobe projection of the arcuate fasciculus that is much smaller or absent in non-human primates (see Figure 4). Moreover, connectivity with the middle temporal gyrus (MTG) was more widespread in the left than in the right hemisphere. Moreover, in humans MTG is found to be one of the most highly connected regions in cerebral cortex (Turken and Dronkers, 2011). This human specialization may be relevant for the evolution of language. Catani et al. (2007) found that the human arcuate fasciculus is strongly lateralized to the left, with quite some variation on the right. On the right, some people lack an arcuate fasciculus, in others it is smaller in size, and only in a minority of the population this fiber bundle is of equal size in both hemispheres. The presence of the arcuate fasciculus in the right hemisphere, correlated with a better verbal memory (but see Gharabaghi et al., 2009, for a non-replication of differences in left and right hemisphere arcuate fasciculi). This pattern of lateralization was confirmed in a study on 183 healthy right-handed volunteers in the age range between 5 and 30 years (Lebel and Beaulieu, 2009). The functionality of the arcuate fasciculus is not limited to single word processing. In a recent paper, Wilson et al. (2012) reported syntactic deficits in patients with primary progressive aphasia after damage to the dorsal tracts but not after damage to the ventral tracts. This suggests that the dorsal tracts including the arcuate fasciculus, are a key component in connecting frontal and temporal regions involved in syntactic processing. This was confirmed in a study by Griffith et al. (2013), although in their case the extreme capsule was equally important.

**Figure 5:**
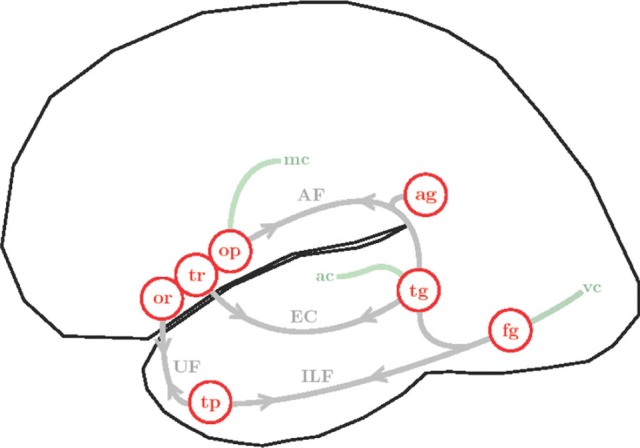
**Simplified illustration of the anatomy and connectivity of the left hemisphere language network**. Cortical areas are represented as red circles: pars orbitalis (or), pars triangularis (tr) and pars opercularis (op) of the LIFC; angular gyrus (ag), superior and middle temporal gyri (tg), fusiform gyrus (fg) and temporal pole (tp). White matter fibers are shown in gray, arrows emphasize bi-directional connectivity: arcuate fasciculus (AF), extreme capsule (EC), inferior longitudinal fasciculus (ILF) and uncinate fasciculus (UC). Interfaces with sensory-motor systems are shown in green: visual cortex (vc), auditory cortex (ac) and motor cortex (mc).

In addition to the arcuate fasciculus, other fiber bundles are important in connecting frontal with temporoparietal language regions (see Figure 5). These include the superior longitudinal fasciculus (adjacent to the arcuate fasciculus) and the extreme capsule fasciculus as well as the uncinate fasciculus, connecting Broca's area with superior and middle temporal cortex along a ventral path (Anwander et al., 2007; Friederici, 2009a,b; Kelly et al., 2010). Figure 5 provides a schematic overview of the more extended connectivity profile of the left perisylvian cortex.

**Figure 6:**
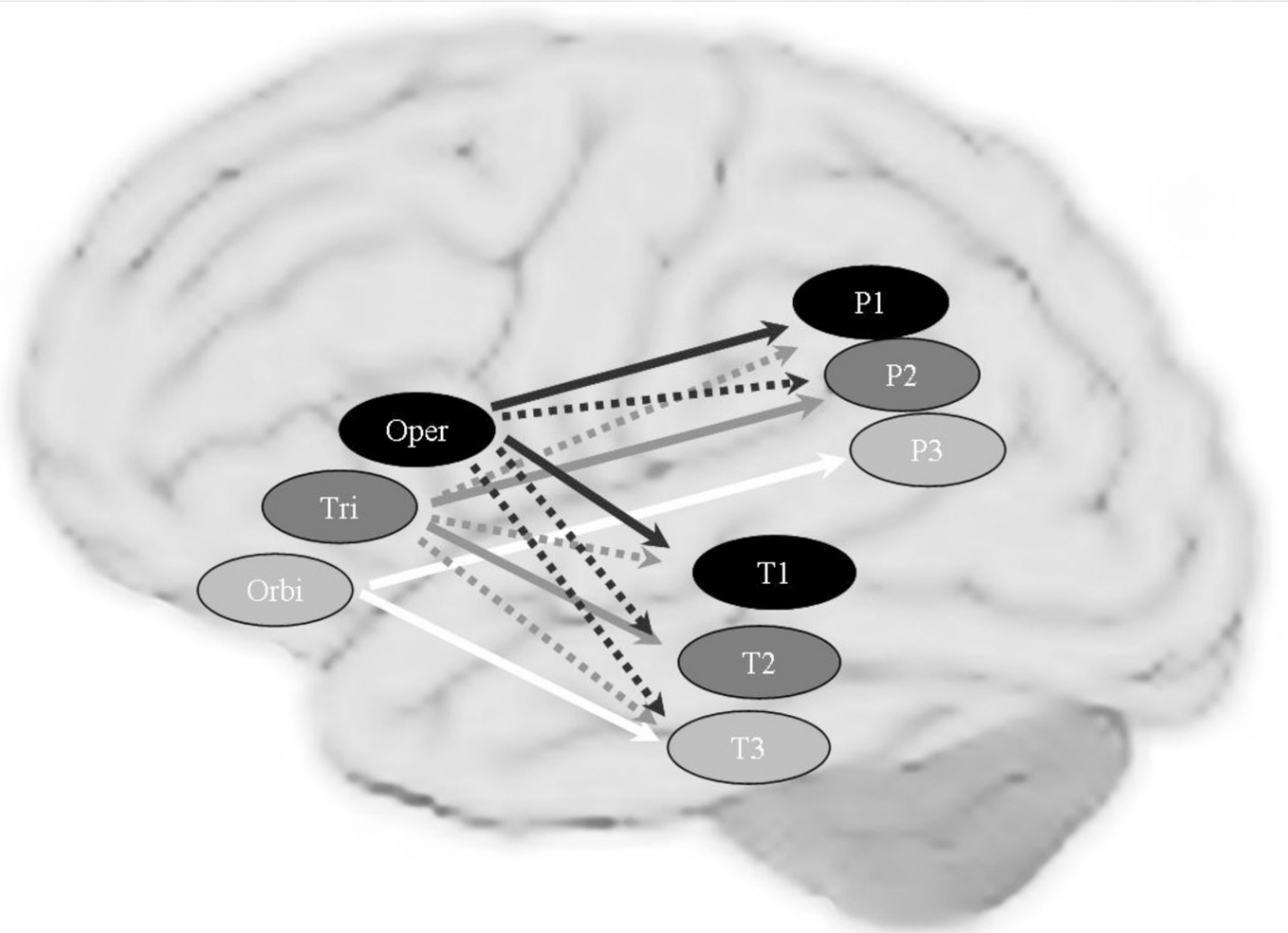
**The topographical connectivity pattern between frontal and temporal/parietal cortex in the perisylvian language networks**. Connections to the left pars opercularis (oper), pars triangularis (tri) and pars orbitalis (orbi) are shown in black, dark gray and white arrows respectively. The solid arrows represent the main (most significant) correlations and the dashed arrows represent the extending (overlapping) connections. Brain areas assumed to be mainly involved in phonological, syntactic and semantic processing are shown in black, dark gray and light gray circles, respectively. P1: Supramarginal gyrus; P3: AG: Angular gyrus; P2: the area between SMG and AG in the superior/inferior parietal lobule; T1: posterior superior temporal gyrus; T2: posterior middle temporal gyrus; P3: inferior temporal gyrus.

DTI is not the only way to trace brain connectivity. It has been found that imaging the brain during rest reveals low-frequency (<0.1 Hz) fluctuations in the fMRI signal. It turns out that these fluctuations are correlated across areas that are functionally connected (Biswal et al., 1995; Biswal and Kannurpatti, 2009). This so-called resting state fMRI can thus be used as an index of functional connectivity. Although both DTI and resting state fMRI measure connectivity, in the case of DTI the connectivity can often be related to anatomically identifiable fiber bundles. Resting state connectivity measures the functional correlations between areas without providing a correlate in terms of an anatomical tract. Using the resting state method, Xiang et al. (2010) found a clear topographical functional connectivity pattern in the left inferior frontal, parietal, and temporal regions (see Figure 6). In the left—but not the right—perisylvian cortex, patterns of functional connectivity obeyed the tripartite nature of language processing (phonology, syntax and semantics). These results support the assumption of the functional division for phonology, syntax, and semantics of the left inferior frontal cortex, including Broca's area. They revealed a topographical functional organization in the left perisylvian language network, in which areas are most strongly connected according to information type (i.e., phonological, syntactic, and semantic). The dorsal pathways might be most relevant for phonological and syntactic processing, while the ventral pathways seem to be strongly, but presumably not exclusively, involved in connecting regions for semantic processing.

## The empirical evidence for the MUC model

We have seen that there is a much more widespread connectivity profile in left perisylvian language cortex than was assumed in the classical WLG model. The MUC model deviates from the classical model in the division of labor between Broca's area, Wernicke's area and adjacent regions. However, the distribution of labor that I propose is not absolute, but embedded and situated in the network skeleton of the language system's neural architecture.

What is the evidence for relative division of labor proposed in the MUC model? Let us consider the syntactic network first. In comparison with phonological and semantic processing, which have compelling bilateral contributions (in contrast to the classical left-hemisphere-only model), syntactic processing seems strongly lateralized to the perisylvian regions in the left hemisphere. Indirect support for a distinction between a memory component (i.e., the mental lexicon) and a unification component comes from neuroimaging studies on syntactic processing. In a meta-analysis of 28 neuroimaging studies, Indefrey (2004) found two regions that were critical for syntactic processing, independent of the input modality (visual in reading, auditory in speech). These two regions for syntactic processing were the left posterior superior/middle temporal gyrus (STG/MTG) and the left inferior frontal cortex. Similar findings have been reported in Kaan and Swaab (2002). The left posterior temporal cortex is known to be involved in lexical processing (Hickok and Poeppel, 2004, 2007; Indefrey and Cutler, 2004; Lau et al., 2006). In connection to the MUC model, this part of the brain might be important for the retrieval of the syntactic frames that are stored in the lexicon. The idea of syntactic frames that specify the possible local syntactic environment of a given lexical item is in line with linguistic and computational approaches that assume syntactic knowledge to be lexically specified (Joshi and Schabes, 1997; Vosse and Kempen, 2000). The Unification Space, where individual frames are connected into a phrasal configuration for the whole utterance, might recruit the contribution of left inferior frontal cortex, (LIFC).

**Figure 7:**
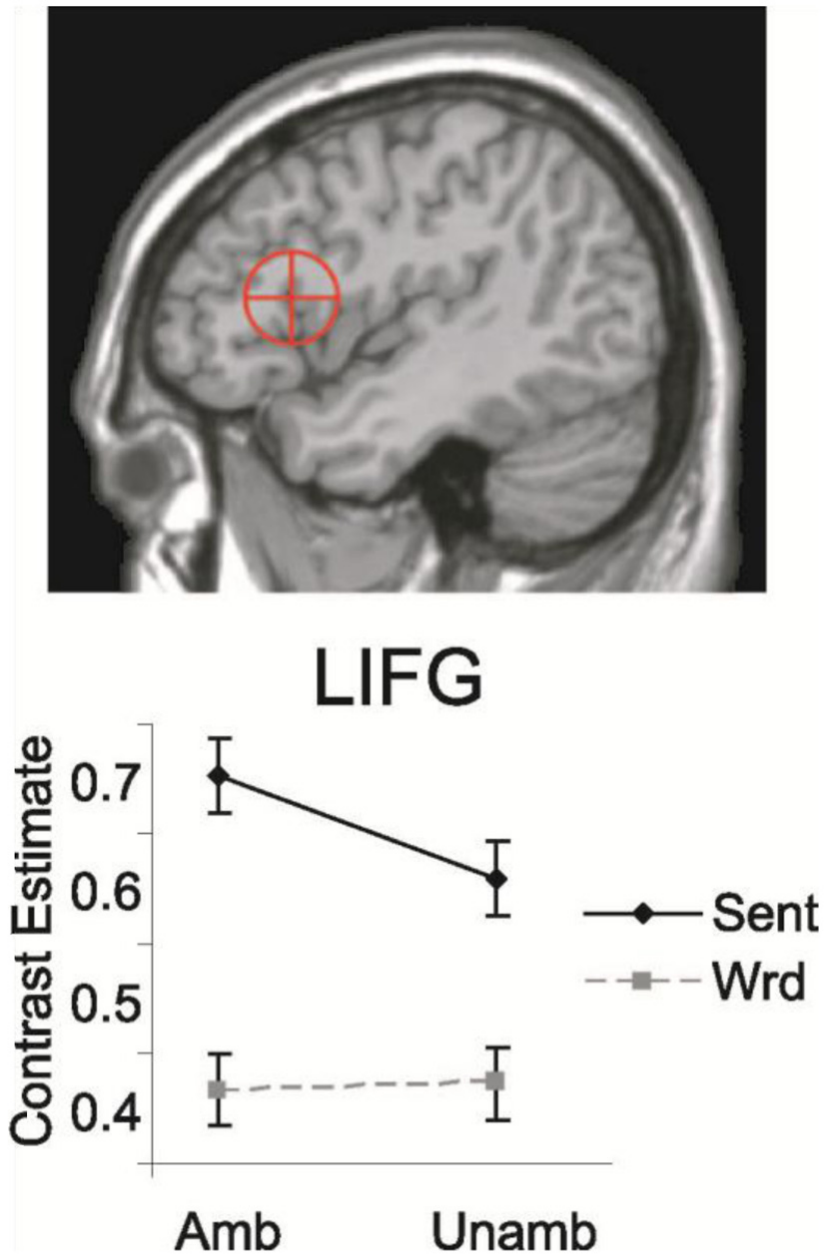
**Mean contrast estimated for LIFC for sentences and word sequences, with (Amb) and without (Unamb) noun-verb ambiguities**. On top the Region of Interest [ROI; 13 mm sphere around coordinates (−44, 19, 14)] is shown. This ROI includes both BA 44 and parts of BA 45 (Snijders et al., 2009).

Direct empirical support for this distribution of labor between LIFC (Broca's area) and temporal cortex was found in a study of Snijders et al. (2009). These authors did an fMRI study in which participants read sentences and word sequences containing word-category (noun-verb) ambiguous words (e.g., “watch”), and the same materials with the unambiguous counterparts of the lexical-syntactic ambiguities. The ambiguous items were assumed to activate two independent syntactic frames, whereas the unambiguous counterparts result in the retrieval of only one syntactic frame. Solely based on a computational model of syntactic processing (Vosse and Kempen, 2000) and the hypothesized contribution of temporal and frontal cortex regions, it was predicted that the regions contributing to the syntactic unification process should show enhanced activation for sentences compared with words, and only within sentences should display a larger signal for ambiguous than unambiguous conditions. The posterior LIFC showed exactly this predicted pattern (see Figure 7), confirming the hypothesis that LIFC contributes to syntactic unification. The left posterior middle temporal gyrus was activated more for ambiguous than unambiguous conditions, as predicted for regions subserving the retrieval of lexical-syntactic information from memory. It thus seems that the left inferior frontal cortex is crucial for syntactic processing in conjunction with the left posterior middle temporal gyrus, a finding supported by patient studies with lesions in these very same regions (Caplan and Waters, 1996; Rodd et al., 2010; Tyler et al., 2011). Presumably these regions are connected via the dorsal pathways.

Next to syntactic unification, there is the need for semantic unification. One aspect of semantic unification is filling the slots in an abstract event schema, where in the case of multiple word meanings for a given lexical item competition and selection are at stake when filling a particular slot in the event schema. As with syntactic unification, the availability of multiple candidates for a slot will increase the unification load. In the case of the lexical-semantic ambiguities there is no syntactic competition, since both readings activate the same syntactic template. For example, the word *bank* has two different readings, but both will activate the NP-template. Increased processing is hence due to unification of meaning instead of syntax. In this case unification is the outcome of competition and selection among two candidates for a slot in the contextually determined event schema.

Semantic processing also recruits a left perisylvian network, albeit with a substantially weaker lateralization profile than syntactic processing. A series of fMRI studies aimed at identifying the semantic processing network. These studies either compared sentences containing semantic/pragmatic anomalies with their correct counterparts (e.g., Kiehl et al., 2002; Friederici et al., 2003; Hagoort et al., 2004; Ruschemeyer et al., 2006) or compared sentences with and without semantic ambiguities (Hoenig and Scheef, 2005; Rodd et al., 2005; Davis et al., 2007). The most consistent finding across all of these studies is the activation of the left inferior frontal cortex (LIFC), more in particular BA 47 and BA 45. For instance, Rodd and colleagues had subjects listen to English sentences such as “There were dates and pears in the fruit bowl” and compared the fMRI response of these sentences to the fMRI response of sentences such as “There was beer and cider on the kitchen shelf.” The crucial difference between these sentences is that the former contains two homophones, i.e., “dates” and “pears,” which, when presented auditorily, have more than one meaning. This is not the case for the words in the second sentence. The sentences with the lexical ambiguities led to increased activations in LIFC and in the left posterior middle/inferior temporal gyrus. In this experiment all materials were well-formed English sentences in which the ambiguity usually goes unnoticed. Nevertheless, very similar results were obtained in experiments that used semantic anomalies.

An indication for the respective functional roles of the left frontal and temporal cortices in semantic unification comes from a few studies investigating semantic unification of multimodal information with language. Using fMRI, Willems and colleagues assessed the neural integration of semantic information from spoken words and from co-speech gestures into a preceding sentence context (Willems et al., 2007). Spoken sentences were presented in which a critical word was accompanied by a co-speech gesture. Either the word or the gesture could be semantically incongruous with respect to the previous sentence context. Both an incongruous word as well as an incongruous gesture led to increased activation in LIFC as compared to congruous words and gestures (for a similar finding with pictures of objects, see Willems et al., 2008). Interestingly, the activation of the left posterior STS was increased by an incongruous spoken word, but not by an incongruous hand gesture. The latter resulted in a specific increase in dorsal premotor cortex (Willems et al., 2007). This suggests that activation increases in left posterior temporal cortex are triggered most strongly by processes involving the retrieval of lexical-semantic information. LIFC, on the other hand, is a key node in the semantic unification network, unifying semantic information from different modalities

From these findings it can be concluded that semantic unification is realized in a dynamic interplay between LIFC as a multimodal unification site on the one hand, and knowledge-specific regions on the other hand. Again it is important to stress that the interplay of these regions is crucial to realize unification.

## A general account of LIFCS role in language processing

So far, we have seen that LIFC plays a central role in syntactic and semantic unification processes, albeit with different activation foci for these two types of unification. It suggests a more general role for LIFC than is claimed by others. For example, proposals have been made that LIFC (Broca's area) has to do with linguistically motivated operations of syntactic movement at the sentence level (Grodzinsky and Santi, 2008), and the processing of hierarchical structures (Friederici et al., 2006). However, there is by now convincing evidence that LIFC also plays a role beneath the phrasal and sentence level. It is found to contribute to decomposition and unification at the word level. Words are not processed as unstructured, monolithic entities. Based on the morpho-phonological characteristics of a given word, a process of lexical decomposition takes place in which stems and affixes are separated. For spoken words, the trigger for decomposition can be as simple as the inflectional rhyme pattern (IRP), which is a phonological pattern signaling the potential presence of an affix (Bozic et al., 2010). Interestingly, words seem to be decomposed by rule; that is to say, decompositional processes are triggered for words with obvious parts (e.g., work-ed) but also for semantically opaque words (e.g., bell-hop), and even non-words with putative parts (e.g., blicket-s, blicket-ed). Decomposing lexical input appears to be a ubiquitous and mandatory perceptual strategy. In a series of fMRI studies on the processing of inflectional morphology, Bozic et al. (2010) have found that LIFC, especially BA 45, subserves the process of morphological decomposition. Intracranial recordings in BA 45 from epileptic patients during presurgical preparation indicate that the same brain area is also involved in the generation of inflected forms during language production (Sahin et al., 2009; see also comments by Hagoort and Levelt, 2009).

The evidence for LIFC involvement in word and sentence level processing in both production and comprehension leads to the question if a general account of its role can be specified. Here is a possible answer. Notwithstanding the division of labor within LIFC, its overall contribution can be characterized in more general terms than hierarchical or even sentence-level processing. Instead, the LIFC is most likely involved in unification operations at the word and sentence level, in connection with temporal and parietal regions that are crucial for memory retrieval (Hagoort, 2005). Compositional and decompositional operations occur at multiple levels and at multiple time slices in the language processing system, but also outside the language system. Any time lexical and other building blocks enter into the process of utterance interpretation or construction, and any time the input string requires decomposition (presumably through analysis-by-synthesis) in order to contact the right lexical representations, LIFC is recruited. The content-specifics of the recruitment are determined by the specific regions and their connectivity profiles, and at specific time slices. As is known for neurons in visual cortex (Lamme and Roelfsema, 2000), the contribution of LIFC may well-vary with time, as a consequence of the different dynamic cortical networks in which it is embedded at different time slices. This fits well with the finding that Broca's region is not language-specific, but also recruited in the service of other cognitive domains, such as music (Patel, 2003) and action (Hamzei et al., 2003), and with the finding that its contribution crosses the boundaries of semantics, syntax, and phonology (Hagoort and Levelt, 2009). Moreover, as recently proposed by Shallice and Cooper (2013), this region might also be involved in the processing of abstract words, since in contrast to concrete words these require “that unification links be made between the arguments of two or more operators”; (Shallice and Cooper, 2013, p. 7).

## The dynamic interplay between memory and unification

Although I have made a connection between functional components of the cognitive architecture for language and specific brain regions, this is an idealization of the real neurophysiological dynamics of the perisylvian language network. Crucially, for language as for most other cognitive functions, the functional contribution of any area or region has to be characterized in the context of the network as a whole, where specialization of any give node is only relative and realized in a dynamic interaction with the other nodes in the network (Mesulam, 1990, 1998). This will be illustrated below on the basis of a new neurophysiological account of the N400, the most well-established ERP effect related to language (Kutas and Hillyard, 1980), and more in particular to semantic unification (but see Dien et al., 2010, for a different account). Similar accounts are to be made for syntactic and phonological unification.

**Figure 8:**
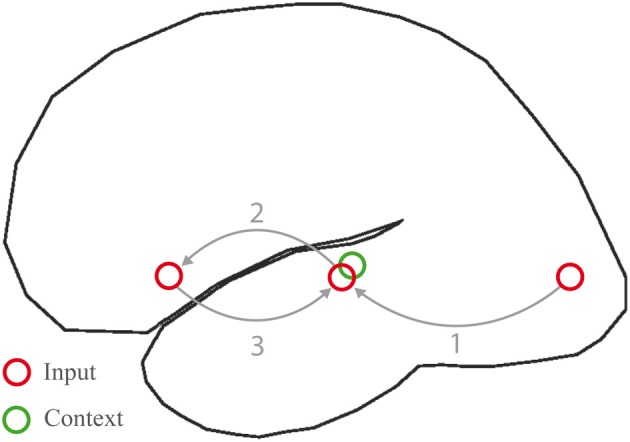
**Processing cycle subserving word meaning comprehension in the left hemisphere language network**. Inputs are conveyed from sensory regions (here visual cortex) to the inferior, middle and superior temporal gyri (1), where lexical information is activated. Signals are hence relayed to the inferior frontal gyrus (2), where neurons respond with a sustained firing pattern. Signals are then fed back into the same regions in temporal cortex from where they were received (3). A recurrent network is thus set up, which allows information to be maintained on-line, a context (green circle) to be formed during subsequent processing cycles, and incoming words to be unified within the context.

The story goes as follows. In posterior and inferior temporal and parietal (angular gyrus) regions, neuronal populations are activated that represent lexical information associated with the incoming word, including its semantic features. From here, neural signals can follow two routes. The first exploits local connectivity within these posterior regions, resulting in a graded activation of neighboring neuronal populations, coding for related lexical-semantic information. Such local spread of activation contributes to setting up a lexical-semantic context in temporo-parietal cortex (Figure 8, green circle), and may underlie priming and pre-activation at short SOAs (Lau et al., 2008). The second route is based on long-distance connections to LIFC, through direct white matter fibers resulting in the selective activation of populations of frontal cortex neurons. These will respond with a self-sustaining firing pattern (see Durstewitz et al., 2000, for a review). Efferent signals in this case can only take the long-range route back. The most parsimonious option is that frontal neurons will send efferent signals back to the same regions in temporo-parietal cortex from where afferent signals were received. This produces another spread of activation to neighboring temporo-parietal regions, which implies that connections representing a given local semantic context will be strengthened. This may be related to priming at longer SOAs, when the contribution of LIFC is also more prominent (Lau et al., 2008). During each word processing cycle the memory (temporo-parietal) and unification (inferior frontal) components interact, by letting activation reverberate through the circuit in Figure 8. Achieving the necessary outcomes for language comprehension may be more or less demanding, depending on how close the relation is between input and context, as we shall see below.

This description of a typical word processing cycle appears to be the simplest possible solution given constraints from brain imaging (the involvement of temporal, parietal, and inferior frontal regions), neuroanatomy (the existence of direct white matter pathways), and neurophysiology (persistent firing of LIFC neurons). However, the proposal is a sketch that requires further elaboration, and a computational implementation that would confer a precise meaning to the envisaged processing steps.

Reverberation in the fronto-temporal circuit might be crucial for basic neurophysiological reasons. Friston (2005) assigns different roles to different neurotransmitters, depending on their decay times. Feedforward connections appear to mediate their post-synaptic effects through fast AMPA and GABA_A_ receptors, and feedback connections are most probably mediated by much slower NMDA receptors. NMDA receptors are relatively frequent in supra-granular layers, where backward connections terminate (Sherman and Guillery, 1998; Sherman, 2007; Kiebel et al., 2008). NMDA-mediated channels may have a role in relaying modulatory effects that are more extended in time (Wong and Wang, 2006). Lisman et al. (1998) have shown that NMDA-receptor mediated EPSPs are critical for the maintenance of information in working memory. They allow a network to maintain its active state without the need for synaptic modification. There is increasing evidence that cortical reverberation by re-entry is important for working memory (Wang, 1999; Fuster, 2009). Baggio and Hagoort (2011) hypothesize that the same is true for language. The feedforward pathways from temporal/parietal cortex to LIFC may be a rapid-decay route requiring NMDA mediated re-entry from LIFC to maintain lexical information active over time, as is essential for multi-word unification.

This neurophysiological account can serve as a basis for a neurocomputational model of the N400. In this proposal the N400 component reflects reverberating activity within the posterior-frontal network during one or perhaps several cycles, as shown in Figure 8. Activity starts building up around 250 ms from word onset, reflecting the summation of post-synaptic currents injected by inferior temporal areas and by neighboring populations in MTG/STG. The direct white matter routes allow for a rapid spread of activation to LIFC. The peak of the N400 coincides with the completion of the cycle; that is with the re-injection of currents into temporal/parietal regions. Across several word-processing cycles, a pattern of neuronal activity emerges in these posterior regions, encoding a local context. This is the result of activation spreading to areas neighboring to those activated by the input during the feedforward sweep, and of a similar process taking place during the feedback from LIFC. This process strengthens learned associations between semantic features. Consider now the case in which semantic relatedness is manipulated, as for instance in “The girl was writing letters when her friend spilled coffee on the tablecloth/paper.” (Baggio et al., 2008). Processing the fragment “The girl was writing letters when her friend spilled coffee on the…” sets up a context, maintained over time by input from LIFC. Semantic features associated with the words *writing* and *letters* are activated (Masson, 1991; Moss et al., 1994; Masson, 1995; Cree et al., 1999; Cree and McRae, 2003; McRae and Ross, 2004; Brunel and Lavigne, 2009). If these include features that contribute to activating the concept of paper, then there will be some overlap between the neuronal populations representing the context and those that selectively respond to the given input, which is to the incoming word *paper*. Such overlap will be smaller for *tablecloth*. The larger the overlap is between context and input, the smaller the amplitude of the scalp-recorded ERP will be. In particular, the incoming word that benefits from a larger overlap with the context (*paper*) results in a smaller N400 compared to the word that leads to a smaller overlap (*tablecloth*). The inverse relation between semantic relatedness and N400 amplitude follows from an inverse relation between the degree of overlap of neuronal sources and the amplitude of scalp-recorded ERPs. The amplitude of any given neuronal generator scales with the size of the contributing population of neurons that are concurrently activated. Under the assumption that there is an N400 unification effect, the increase in the N400 amplitude as a function of unification load can be explained as follows. Neuronal populations in LIFC (coding for the current non-local context), upon receiving input from temporal/parietal cortex, start firing in a sustained manner, and inject currents back into the same regions from where signals were received. In this way transient links are dynamically established between semantic types for which temporal and parietal cortex might be the hubs (convergence zones of distributed representations). Regardless of whether the N400 effect is driven by pre-activation or by unification, the theory is consistent with the finding that some of the strongest neuronal generators of N400 are localized in the left middle and superior temporal cortex. This is where most afferent signals are projected: (1) from peripheral areas via inferior temporal cortex during early processing stages (~200 ms); (2) through local connectivity in MTG/STG due to spreading activation from input-selective populations to neighboring temporal areas; (3) from LIFC during the feedback that supports unification and the on-line maintenance of context. LIFC may show a comparatively smaller net effect of post-synaptic currents over shorter time intervals, possibly due to fewer signals re-injected through local connectivity in LIFC itself, but a stronger activation (as revealed by metabolic measures) over longer time periods, due to the persistent firing patterns produced by LIFC neurons. This could explain why MEG/EEG source analyses may fail to reveal significant contributions of LIFC, whereas fMRI does show a strong response in LIFC. Also, the time-locking of neuronal responses appears to be sharper in posterior temporal cortex than in inferior frontal areas (Liljeström et al., 2009). Activity in LIFC is presumably relatively insensitive to the onset and offset times of the stimuli, and is rather a self-sustaining state which is relatively unaffected by trial-to-trial variation. In contrast, bottom-up activation in MTG/STG and adjacent regions may have tighter deadlines, partly due to the proximity to sensory areas.

This account of the N400 (for further details, see Baggio and Hagoort, 2011) is consistent with available anatomical and functional data, as well as with recent accounts as proposed by Kutas and Federmeier in their review of 30 years N400 research (Kutas and Federmeier, 2011) and by Nieuwland et al. (Nieuwland et al., 2010). It explains the N400 as resulting from the summation of currents injected by frontal into temporal/parietal areas (unification) with currents that are already circulating within the latter regions due to the local spread of activation to neighboring neuronal populations (pre-activation). Hence, pre-activation and unification do not result in mutually exclusive accounts of the N400. In real-time language processing access, selection, pre-activation and unification are all part of a word processing cycle; that is, a continuous pattern of neuronal activity unfolding over time within a distributed cortical network.

## Attentional control

**Figure 9:**
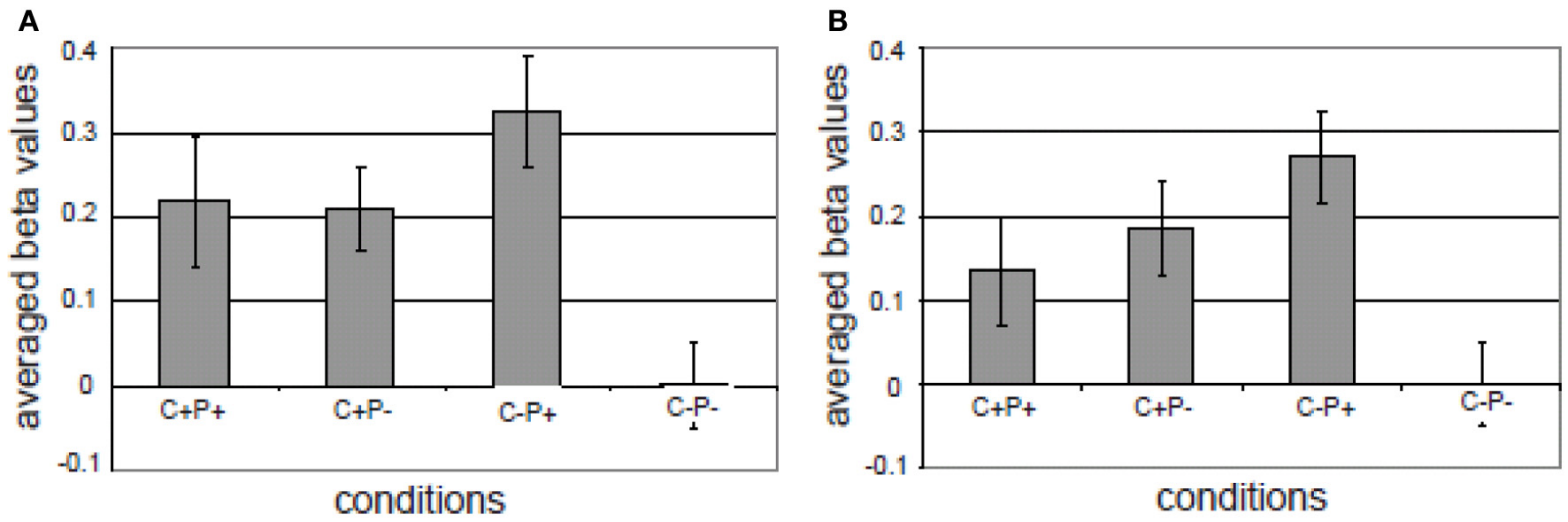
**Different activations in the four conditions in (A) left superior/inferior parietal cortex; (B) right superior/inferior parietal and right supramarginal region**. The gray bars represent the averaged beta values of four conditions in the ROI (the activation in the C–P– condition was taken as an arbitrary zero in the diagram). The vertical lines indicate the standard error for each condition. C+P+: Congruent, with pitch accent; C+P–: Congruent, without pitch accent; C–P+: Incongruent, with pitch accent; C–P–: Incongruent, without pitch accent (from Kristensen et al., 2012).

The third component in the MUC model is referred to as Control. One form of control is attentional control. In classical models of sentence comprehension—of either the syntactic-structure-driven variety (Frazier, 1987) or in a constraint-based framework (Tanenhaus et al., 1995)—the implicit assumption is usually that a full phrasal configuration results and a complete interpretation of the input string is achieved. However, oftentimes the listener interprets the input on the basis of bits and pieces that are only partially analyzed. As a consequence, the listener might overhear semantic information (cf. the *Moses illusion*; Erickson and Mattson, 1981; Wang et al., 2011) or syntactic information (cf. the *Chomsky illusion*; Wang et al., 2012). To the question “How many animals of each kind did Moses take on the ark?,” listeners often answer “two,” without noticing that it was Noah who was in command of the ark, and not Moses. It was found that likewise syntactic violations might not trigger a brain response if they are in a sentence constituent that provides no new information (Wang et al., 2012). Ferreira et al. (2002) introduced the phrase “good-enough processing” to refer to the listeners' and readers' interpretation strategies. In a good-enough processing context, linguistic devices that highlight the most relevant parts of the input might help the listener/reader in allocating processing resources optimally. This aspect of linguistic meaning is known as “information structure” (Halliday, 1967; Chafe, 1976; Buring, 2007; Krifka, 2007). The information structure of an utterance essentially focuses the listener's attention on the crucial (new) information in it. In languages such as English and Dutch, prosody plays a crucial role in marking information structure. For instance, in question-answer pairs, the new or relevant information in the answer will typically be pitch accented. After a question like “What did Mary buy at the market?,” the answer might be “Mary bought VEGETABLES” (accented word in capitals). In this case, the word *vegetables* is the focus constituent, which corresponds to the information provided for the Wh-element in the question. There is no linguistic universal for signaling information structure. The way information structure is expressed varies within and across languages. In some languages it may impose syntactic locations for the focus constituent, in other languages focus-marking particles are used, or prosodic features like phrasing and accentuation (Kotschi, 2006; Miller et al., 2006). In a recent fMRI study (Kristensen et al., 2012), we tested the idea that pitch accent, which in Dutch is used to mark certain information as focus, recruits the attention network in the service of more extended processing of the most relevant information. In our study, we first localized the attention network in an auditory, non-verbal attention task. This task activated, as expected, bilateral superior and inferior parietal cortex. In the language task participants were listening to sentences with and sentences without semantic-pragmatic anomalies. In half of the cases these anomalies and their correct counterparts were in focus as marked by a pitch accent, in the other half of the cases they were not. The results showed an interaction in bilateral inferior parietal regions between prosody (pitch accent) and congruence (see Figure 9): for incongruent sentences there was a larger activation if the incongruent words carried a focus marker (i.e., the pitch accent).

Overall, the activation overlap in the attention network between the localizer task and the sentence processing task indicated that marking of information structure modulated a domain general attention network. Pitch accent signaled the saliency of the focused words and thereby recruited attentional resources for extended processing. This suggests that languages might have developed built-in linguistic devices (i.e., focus markers) that trigger the recruitment of the attention system to safeguard against the possibility that the most relevant information might go unnoticed. This provides one example of the interaction between a general demand/control system (Fedorenko et al., 2012) and the core components of the language network.

## Beyond the core regions

**Figure 10:**
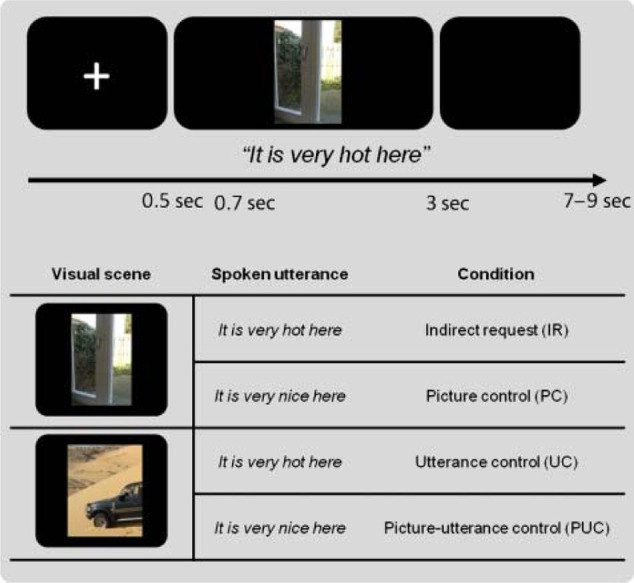
**Illustration of the conditions and the presentation parameters of the fMRI stuy on indirect requests (IR)**. The top half shows the time course of presentation. On each trial a fixation cross was presented for 500 ms, followed by a visual scene. The utterance was presented auditorily, 200 ms after picture onset. Each trial lasted 3 sec. The bottom half depicts one item in the four conditions. For further details, see (van Ackeren et al., 2012).

So far I have implicitly assumed that decoding the meaning of an utterance is what language comprehension is about. However, while this might be a necessary aspect, it cannot be the whole story. Communication goes further than the exchange of explicit propositions. In essence the goal of the speaker is to either change the mind of the listener, or to commit the addressee to the execution of certain actions, such as closing the window in reply to the statement “It is cold here.” In other words, a theory of speech acts is required to understand how we get from coded meaning to inferred speaker meaning (cf. Grice, 1989; Levinson, 2013a, b). We have recently shown that the inference of speaker meaning requires the contribution of the Theory of Mind (ToM) network, including the temporo-patietal junction (TPJ) and medial prefrontal cortex (mPFC). In one such study (van Ackeren et al., 2012) we presented subjects with sentences in the presence of a picture. In one condition the sentence in combination with the picture could be interpreted as an indirect request for action. For example, the utterance “It is hot here” combined with a picture of a door is likely to be interpreted as a request to open the door. However, the same utterance combined with the picture of a desert will be interpreted as a statement (see Figure 10, for a specification of the conditions).

**Figure 11:**
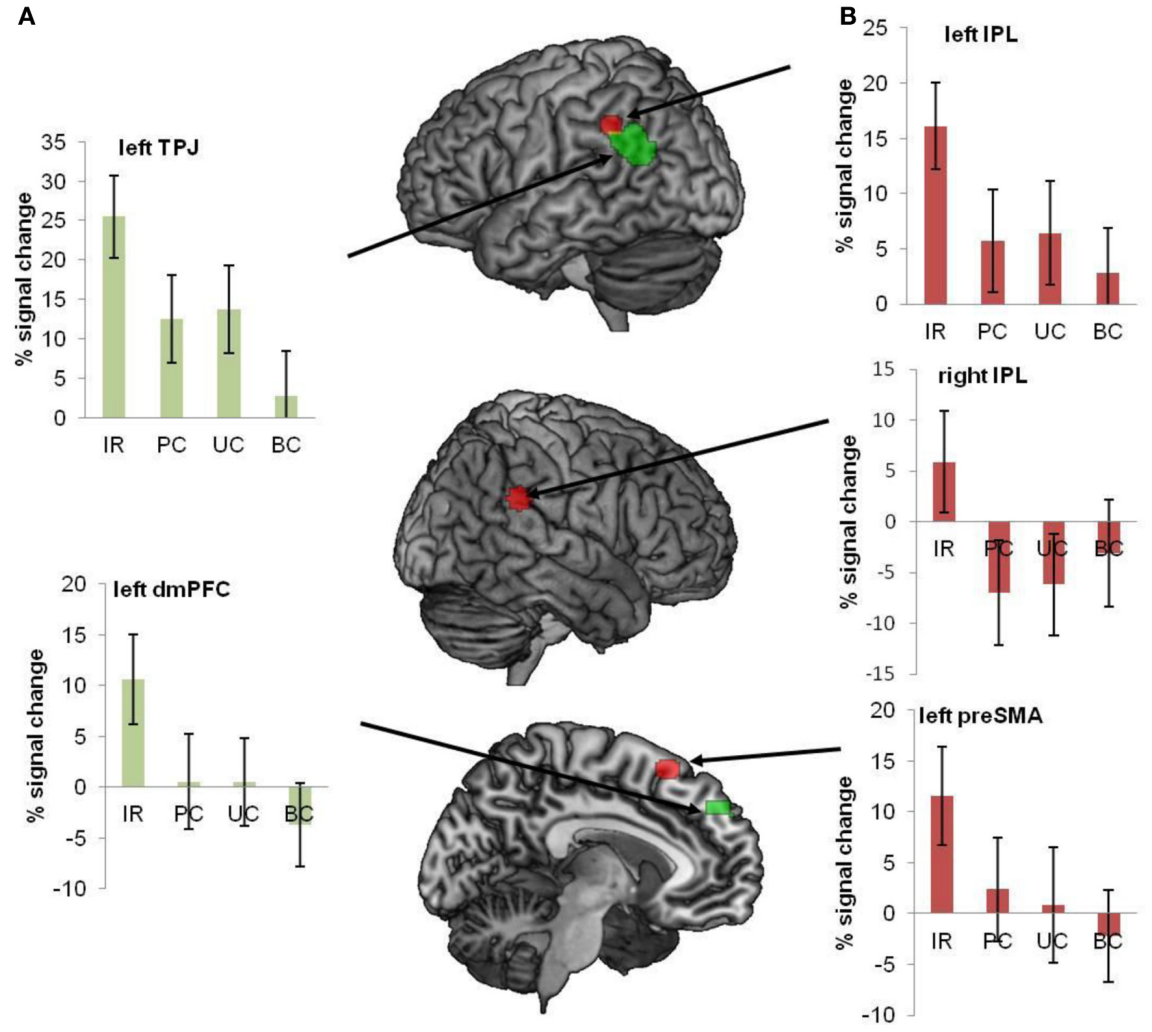
**Regions of interest were interrogated with respect to the conditions IR, PC, UC, and BC**. The image shows all ROI's, superimposed on a brain template. The bar diagrams illustrate mean percent signal change for each condition. The error bars depict the standard error. **(A)** Green ROIs show regions from the ToM localizer (mPFC and TPJ). **(B)** Red ROIs refer to regions that were activated during action execution (pre-SMA and bilateral IPL) (van Ackeren et al., 2012).

Van Ackeren et al. found that sentences in the indirect request (IR) condition activated the ToM network much stronger than the very same sentences in the three control conditions (see Figure 11). The conclusion is that regions for sensorimotor simulation are not sufficient for deriving speaker meaning, which is of the essence in ordinary language comprehension. The pragmatics of language interpretation in context seems to require the inferential machinery instantiated in the ToM network. A similar result was obtained in a recent fMRI study on conversational implicatures in indirect replies (Question: “Did you like my presentation?,” Answer: “It is hard to give a good presentation”; Bašnáková et al., 2013). Interestingly, van Ackeren et al. (2012) also found action-related regions more strongly activated in the IR condition. The indirect request for action seems to induce action preparation automatically, even in sentences that do not contain any action words. For a summary of the results, see Figure 11.

## Beyond the classical model

I have outlined the contours of a neurobiological model of language that is a substantial deviation of the classical WLG model, which was mainly based on lesion and patient data. Three major deviations are worth highlighting: (1) the connectivity of the language cortex in left perisylvian regions is much more extended than proposed in the classical model and is certainly not restricted to the arcuate fasciculus; (2) the distribution of labor between the core regions in left perisylvian cortex is fundamentally different than proposed in the classical model. It assumes shared circuitry for core aspects of language production and comprehension, which both recruit temporal/parietal regions for retrieval of linguistic information that is laid down in memory during acquisition, and unification of building blocks into utterances or interpretations that are constructed on-line. Unification “enables words to cooperate to form new meanings” (Nowak, 2011, p. 179). (3) The operation of language in its full glory requires a much more extended network than what the classical model contained, which was mainly based on evidence from single word processing. The basic principle of brain organization for higher cognitive functions is that these are based on the interaction between a number of neuronal circuits and brain regions that support the different contributing functional components. These circuits are not necessarily specialized for language, but nevertheless need to be recruited for the sake of successful language processing. One example is the attention network that might be triggered into operation by specific linguistic devices to safeguard against missing out on the most relevant (new, focused) information in the language input. The other example is the ToM network that seems crucial for designing our utterances with knowledge of the listener in mind and, as a listener, to make the step from coded meaning to speaker meaning. Finally, as I sketched in the account of the N400, the system is dynamic in contrast to what might be implicitly suggested by the static pictures of the neuronal infrastructure for language. The specific contribution to information processing of any area is dependent on the input it receives at a certain time-step, which itself depends on the computational environment in which it is embedded (see Petersson and Hagoort, 2012, for a formal account). It seems clear that a dynamical systems approach based on spiking neural networks is necessary to grasp the full spatiotemporal profile of language processing.

### Conflict of interest statement

The author declares that the research was conducted in the absence of any commercial or financial relationships that could be construed as a potential conflict of interest.
